# Quality of Life of Patients With Inflammatory Bowel Disease in Bangladesh

**DOI:** 10.7759/cureus.39929

**Published:** 2023-06-04

**Authors:** Chanchal Kumar Ghosh, Sumona Islam, Nowrin Tabassum, Syed Arafat Mohiuddin, Md. Mosarrof Hossain, Aditi Sarkar, Amit Bari

**Affiliations:** 1 Department of Gastroenterology, Bangabandhu Sheikh Mujib Medical University, Dhaka, BGD; 2 Department of Nephrology, Kidney Foundation Hospital and Research Institute, Dhaka, BGD

**Keywords:** utility index, eq-5d-5l, health-related quality of life (hrqol), ulcerative colitis, crohn's disease

## Abstract

Introduction

The importance of maintaining quality of life in managing inflammatory bowel disease (IBD) has increased in recent years. However, there is a lack of studies examining the health-related quality of life (HRQoL) of IBD patients in Bangladesh.

Methodology

This cross-sectional study was carried out in the IBD clinic, Bangabandhu Sheikh Mujib Medical University (BSMMU) from 2020 to 2022. Data were collected from both ulcerative colitis (UC) and Crohn’s disease (CD) patients. HRQoL was recorded on the EuroQol 5 Dimension 5 Level (EQ-5D-5L) questionnaire. Statistical analysis was done by Statistical Analysis Software (SAS, SAS Institute, Cary, NC).

Results

The mean age was 36.3 years. The majority of the patients were male and had low incomes. People with more monthly income, more frequent relapse, extraintestinal involvement, and moderate to severe disease had lower utility index (p = 0.01, 0.01, 0.0004, and <0.0001, respectively). Among the five individual components, only usual activity was lower in UC patients (p = 0.03); all the other components and consequently the overall utility index did not vary between UC and CD. The visual analog scale (VAS) score seemed to be comparable in UC and CD patients.

Conclusion

In more severe and frequently relapsing cases of IBD, the utility index representing HRQoL was found to be lower. Comparatively, the HRQoL was mostly similar between patients with UC and CD. Additionally, the mean utility score in IBD patients was higher than that observed in patients with type 2 diabetes mellitus in Bangladesh.

## Introduction

Inflammatory bowel disease (IBD) is a chronic inflammatory disorder of the gastrointestinal tract, associated with inflammation of the intestinal wall along with a multitude of systemic complications [1]. It typically encompasses two main disease entities: ulcerative colitis (UC) and Crohn’s disease (CD). Being multifactorial in nature, as far as our pathophysiological understanding has progressed, a complex interaction between immunological, genetic, and environmental factors leading to a dysregulated immune response against gut microbiota is responsible for the disease [2].

The global age-standardized prevalence rate increased from 79.5 to 84.3 per 100,000 population from 1990 to 2017, translating to about 6.8 million people living with IBD globally in 2017. The prevalence was the highest in North America and lowest in the Caribbean region [3]. Data regarding IBD from South Asia, especially Bangladesh are scarce.

There have been remarkable innovations and advances in the medical and surgical management of IBD. This has led to a steady increase in life expectancy in IBD patients [4]. With this improved management and increased life expectancy, more and more patients are living with IBD, adding to the global IBD burden. For many years, the goal of IBD treatment has been to improve symptoms, decrease inflammatory markers, achieve healing of ulcers on endoscopy, and prevent complications. But the chronic nature of the disease, along with its relapsing and remitting course has made maintaining the quality of life a more relevant goal for the management of IBD [5].

The EuroQol 5 Dimension 5 Level (EQ-5D-5L) questionnaire has been used widely to measure the quality of life of the IBD population across different countries, including the United States, several countries across Europe, Saudi Arabia, Korea, China, Singapore, etc. [6-10]. EuroQol 5 Dimension (EQ-5D), introduced in 1990, was later refined and renamed to EQ-5D-5L. It is a generic instrument that measures the quality of life over five dimensions: mobility, self-care, usual activities, pain/discomfort, and anxiety/depression. It also includes a visual analog scale (VAS) [11,12].

No studies have been conducted in Bangladesh to examine the quality of life of patients with IBD. The aim of this study is to examine the quality of life of Bangladeshi IBD patients.

## Materials and methods

Study design and patient selection

This was a cross-sectional study that was carried out in the IBD clinic, Department of Gastroenterology, Bangabandhu Sheikh Mujib Medical University (BSMMU) from October 2020 to September 2022. UC and CD patients both diagnosed in the in-patient setting in BSMMU and referred from other centers were followed up in the clinic. Diagnosed IBD patients with age more than 18 years, who provided informed written consent were included in the study.

Data collection and measurement of quality of life

Baseline demographic data, clinical parameters, and laboratory investigations were collected on pre-tested questionnaires through history, physical examination, and laboratory investigations. Quality of life was recorded on the EQ-5D-5L questionnaire [13]. The questionnaire comprises five dimensions: mobility, self-care, usual activities, pain/discomfort, and anxiety/depression. Each dimension offers five levels, ranging from no problems to extreme problems. Participants indicate their health state by selecting the most suitable statement for each dimension, resulting in a five-digit number representing their health condition. Index scores are calculated based on surveys conducted in various countries using time trade-off methods among the general population [13]. In the absence of a validated value set for Bangladesh, the Indian version was adopted [14]. Higher index scores, which fall between -1 and 1, indicate a better quality of life.

The EuroQol Visual Analog Scale (EQ-VAS) measures patients' subjective perception of their health on a visual analog scale. The scale features two endpoints labeled "best imaginable health state" and "worst imaginable health state" [13]. In this study, higher values on the scale, ranging from 0 to 100 points, indicate a higher quality of life.

Statistical analysis

Computer-based statistical analysis was carried out with appropriate techniques and systems. All data were recorded systematically in the pre-formed data collection form. A descriptive analysis was performed for the demographic and clinical characteristics and results were presented as mean ± standard deviation or median ± interquartile range (IQR) as appropriate for quantitative variables and numbers for qualitative variables. The individual components of the EQ-5D-5L questionnaire were analyzed both descriptively and in association with IBD activity scores and clinical and biochemical parameters. Statistical analyses were performed using Statistical Analysis Software (SAS) Studio version (SAS Institute, Cary, NC). The difference in means or percentages of different variables was calculated using either the chi-square test for nominal variables or the unpaired Student's t-test for numerical variables. P-values less than 0.05 were considered significant.

## Results

The study enrolled a total of 155 patients, comprising 86 patients with UC and 69 with CD. The mean age in the UC group was 36 years, while in the CD group, it was 36.6 years. Most of the patients were male and had a monthly income of less than 20,000 takas (around 200 dollars). Notably, the CD group had more male patients and a higher proportion of current and past smokers. Detailed demographic characteristics are shown in Table 1.

**Table 1:** Demographic characteristics UC: ulcerative colitis; CD: Crohn’s disease.

Variables	Categories	UC (n = 86)	CD (n = 69)	p-value
Age (mean)		36	36.6	0.78
Gender	Male	49 (57%)	56 (81.1%)	0.009
	Female	37 (43%)	13 (18.9%)	
Residence	Rural	46 (53.5)	36 (49.3%)	0.65
	Urban	32 (37.2)	32 (43.8%)	
	Semi-urban	8 (9.3%)	5 (6.9%)	
Monthly income (taka)	<20,000	49 (57%)	36 (50%)	0.47
	20,000-40,000	28 (32.5%)	24 (33.3%)	
	>40,000	9 (10.5%)	12 (16.7%)	
Smoking status	Smoker	1 (1.2%)	3 (4.2%)	0.03
	Past smoker	10 (11.8%)	18 (25%)	
	Non-smoker	74 (87%)	51 (70.8%)	

The overall utility index according to the demographic, clinical, and biochemical profile of all patients is presented in Table 2.

**Table 2:** Distribution of the utility index according to demographic profile

Variables	Categories	Utility index	p-value
Gender	Male	0.77	0.41
	Female	0.72	
Age group (years)	<30	0.78	0.57
	30-50	0.74	
	>50	0.7	
Residence	Rural	0.76	0.66
	Urban	0.73	
	Semi-urban	0.8	
Monthly income (taka)	<20,000	0.76	0.01
	20,000-40,000	0.8	
	>40,000	0.56	
Duration of disease	<1 year	0.78	0.88
	1-5 years	0.745	
	>5 years	0.747	
Relapse of disease	Infrequent	0.817	0.01
	Frequent (>= 2)	0.683	
	Continuous	0.61	
Extraintestinal involvement	Present	0.61	0.0004
	Absent	0.807	
Hemoglobin (gm/dl)	<10.5	0.809	0.13
	>10.5	0.725	
C-reactive protein (mg/dl)	Normal	0.739	0.6
	High	0.766	
Fecal calprotectin (ug/g)	Normal	0.773	0.32
	High	0.722	
Serum albumin (g/dl)	Normal	0.821	0.09
	Low	0.725	

The study found no significant variation in the utility index based on age group, sex, or residence (p = 0.41, 0.57, and 0.66, respectively). However, patients with a monthly income exceeding 40,000 takas appeared to have a lower utility index (p = 0.01). Those who experienced more frequent relapses and extraintestinal involvement had a lower utility index (p = 0.01 and 0.0004, respectively). The baseline hemoglobin, C-reactive protein (CRP), fecal calprotectin, and serum albumin levels did not appear to affect the utility index. The utility index according to the location of disease and severity is shown in Table 3.

**Table 3:** Distribution of the utility index according to disease location in UC and CD patients UC: ulcerative colitis; CD: Crohn's disease.

Variables	Categories	Utility index	P-value
Ulcerative colitis	Proctitis	0.892	0.41
	Left-sided colitis	0.794	
	Pan-colitis	0.75	
	Pouchitis	0.787	
Crohn's disease	Terminal ileum	0.664	0.36
	Colon	0.646	
	Ileocolon	0.822	
	Isolated upper GI	0.719	
Disease type (both UC and CD)	Remission	0.87	<0.0001
	Mild	0.82	
	Moderate	0.59	
	Severe	0.64	

**Figure 1:**
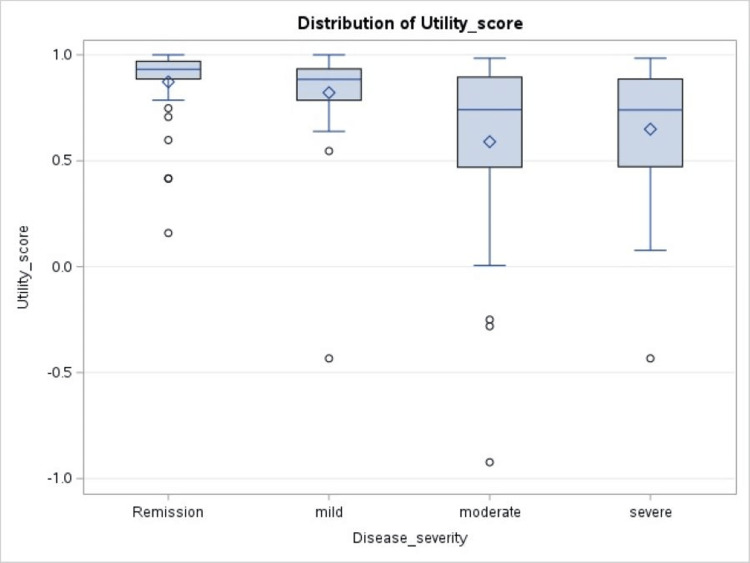
Box and whisker plot showing variation in mean utility score according to disease severity The mean score for patients with remission = 0.87, mild disease = 0.82, moderate disease = 0.59, and severe disease = 0.64. P-value = <0.0001.

Disease severity was assessed by partial Mayo score in the case of UC and Harvey-Bradshaw Index in the case of CD. The location of the disease did not appear to affect the utility index in patients with either UC or CD. But moderate to severe disease had a lower utility index (p < 0.0001; Figure 1).

**Figure 2:**
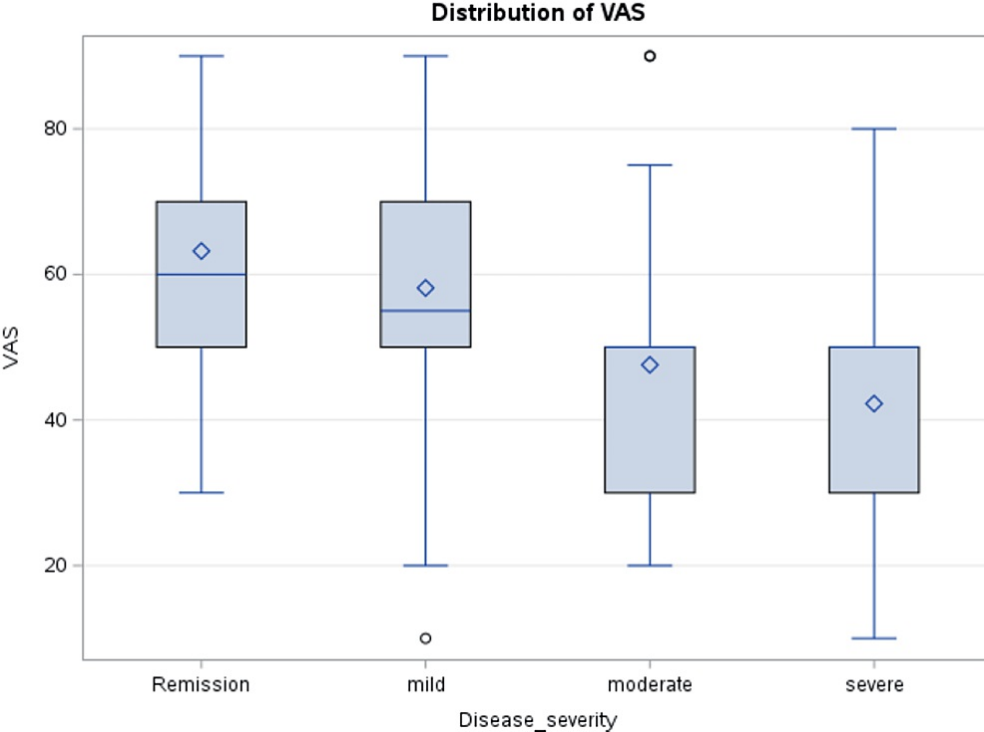
Box and whisker plot showing variation in visual analog scale (VAS) according to disease severity The mean score for patients with remission = 63, mild disease = 58, moderate disease = 47, and severe disease = 42. P-value = <0.0001.

VAS score was also low in moderate to severe disease (p < 0.0001) (Figure 2).

Table 4 displays the utility index in CD based on disease behavior and the presence of a perianal fistula. Patients with fistulating disease and perianal disease exhibited a lower utility index (p = 0.002 and 0.007, respectively).

**Table 4:** Utility index in Crohn's disease based on disease behavior and the presence of perianal disease

Variables	Categories	Utility score	P-value
Disease behavior	Nonstricturing, nonpenetrating	0.8	0.002
	Stricturing	0.733	
	Fistulating	0.227	
	Stricturing and fistulating	0.721	
Perianal disease	Yes	0.529	0.007
	No	0.78	

Table 5 shows the comparison of the five individual components of the utility index, overall index, and VAS of UC and CD patients. Among the five components, only usual activity was lower in UC patients (p = 0.03). All the other components and consequently the overall utility index did not vary between UC and CD patients. The VAS also seemed to be comparable in UC and CD patients.

**Table 5:** Comparison of individual quality of life components, utility index, and VAS in UC and CD patients UC: ulcerative colitis; CD: Crohn's disease; VAS: visual analog score.

Dimensions	UC	CD	P-value
Mobility (MO)	1.7	1.87	0.3
Usual activity (UA)	1.482	1.797	0.03
Self-care (SC)	1.663	1.841	0.27
Pain or discomfort (PD)	1.687	1.841	0.28
Anxiety or depression	2.602	2.565	0.82
Overall utility index	0.79	0.705	0.09
VAS	54.7	52.3	0.42

## Discussion

Different tools have been used to assess the HRQoL of IBD patients over the years. Although EQ-5D-5L is not IBD-specific, it has been used in the general population and in a wide variety of chronic diseases across numerous countries for a long time and has been widely validated and refined over time [12,13,15]. This together with its ease of use has made it a popular choice for assessing HRQoL in IBD patients, especially in recent times. Several studies have used it in the IBD population with good results [6-10].

The development of a population-specific time trade-off value set is of great importance to correctly analyze the collected data. Unfortunately, no such tool was available for the Bangladeshi population and prior studies from this country that collected HRQoL data using the EQ-5D-5L questionnaire mostly relied on the value set from the UK population to calculate the unified utility score. These studies were all focused on determining the HRQoL of the diabetic population in this country [16-18]. To our knowledge, there has been no study looking into the quality of life of IBD patients in Bangladesh.

We had a relatively younger group of patients compared to the other studies we came across looking into HRQoL in IBD [6,7,19]. The mean age of our UC patients was 36 years, and it was 36.6 years for CD patients. The CD group had 76.7% male patients, much higher than the UC group. This might explain the increased percentage of smokers and ex-smokers in the CD group, as it is the usual trend in Bangladesh [20]. Other demographic characteristics did not seem to vary significantly between the two groups.

The overall calculated mean utility score was 0.75: 0.77 in males and 0.72 in females. This score varied widely across studies and according to disease activity and level of remission, ranging from 0.66 to 0.92. However, our mean utility score was lower than most [6,7,9,21]. The score did not vary significantly according to gender, age group, and residence. But people in the higher income category, earning more than 400 USD a month, seemed to have a lower utility score (p = 0.01). A similar pattern was observed in a study on Chinese CD patients, where patients with more healthcare-related expenditures had a lower score [9]. Since no study looking at the HRQoL of the general population using the EQ-5D-5L questionnaire could be found in Bangladesh, we compared our result with HRQoL data from people with type 2 diabetes mellitus (DM). Our mean score of 0.75 was higher than the mean score of diabetic patients from several studies, with a mean of 0.62 [17,18]. Both those studies were population-based, dealing with DM patients with unknown treatment and follow-up status. Whereas our patients were on treatment and follow-up, which could account for this discrepancy. Previous studies had different findings in this regard. While two studies found that the utility score was lower in IBD patients compared to the general population [21,22]. In contrast, several studies across Europe found no difference in the HRQoL between the IBD and the general population [19,23]. A study in Belgium that directly compared the HRQoL in patients with type 2 DM, however, found no significant difference between the two groups [24].

Duration of the disease did not seem to affect the utility score (p = 0.88), illustrating the chronic nature of the disease. At the same time, it emphasizes the fact that, with proper effort, IBD can be effectively managed regardless of the duration. In our study, clinical parameters seemed to strongly affect the score. Patients with more relapses and extra-intestinal involvement had a lower score (p = 0.01 and 0.0004, respectively). This finding resonated with a study performed on the IBD population in Singapore [10]. Interestingly, however, laboratory investigations that are usually used to assess or predict disease status did not seem to affect the HRQoL in our study. Fecal calprotectin, CRP, serum albumin, and hemoglobin level were not associated with the utility scores. One study that looked at CRP also did not find it to be a good predictor of HRQoL [10]. This again emphasizes the need for checking and focusing on HRQoL status to assess the patient’s status on presentation and follow-up besides the clinical and biochemical parameters traditionally used.

Disease activity strongly predicted HRQoL. In UC patients, we used the partial Mayo score to categorize disease activity and remission, and in CD patients, the Harvey-Bradshaw Index was used to categorize disease activity and remission. Patients with moderate and severe disease had much lower scores compared to patients with milder disease or in remission (p < 0.0001). Most of the studies that looked at HRQoL in IBD patients had similar observations [6,7,9,21,25]. Involvement of different parts of the intestine on endoscopy did not seem to affect the quality of life in our UC or CD patients (p = 0.41 and 0.36, respectively). One study done on CD patients, however, found that patients with lesions on the ileum had higher utility scores [9].

When comparing UC and CD patients, they did not seem to differ in terms of their overall utility score in our study (p = 0.09). When we looked at the five individual components, only the usual activity score was lower in UC than in CD patients (p = 0.03), and the others were similar. Previous studies that compared HRQoL in UC and CD patients found similar results in terms of the over-utility score [10,23]. The VAS score also did not seem to differ between UC and CD in our study (p = 0.42).

To our knowledge, this is the first study that has looked at and compared the HRQoL in UC and CD patients in Bangladesh. This study will inspire future IBD-related research and will highlight the need to emphasize HRQoL in IBD management in Bangladesh while reiterating why a national IBD guideline is so necessary. Our study is based on out-patients, making milder diseases more common. Since very few centers systematically follow up and register IBD patients in Bangladesh, we had to rely on a single-center design with a relatively small sample size.

## Conclusions

HRQoL is the modern yardstick of well-being in IBD patients. In our study, we found it to be very effective in assessing status. The overall utility score of IBD patients using the EQ-5D-5L questionnaire was higher than previous studies done in Bangladesh on type 2 DM patients. Frequent relapses, extraintestinal involvement, and higher disease activity seemed to mostly lead to lower scores. HRQoL did not seem to differ between UC and CD patients. Future larger-scale multi-centered studies with follow-ups, using different instruments, should be done in Bangladesh to properly assess the HRQoL of IBD patients and the factors affecting it.
